# Vitamin D Supplementation in Heart Failure—Confusion Without a Cause?

**DOI:** 10.3390/nu17111839

**Published:** 2025-05-28

**Authors:** Zofia Kampka, Dominika Czapla, Wojciech Wojakowski, Agata Stanek

**Affiliations:** 1 Department of Cardiology and Structural Heart Diseases, Medical University of Silesia, 45/47 Ziołowa St., 40-635 Katowice, Poland; czapladominika7@gmail.com (D.C.); wwojakowski@sum.edu.pl (W.W.); 2 Upper-Silesian Medical Center, 45/47 Ziołowa St., 40-635 Katowice, Poland; 3 Department of Internal Medicine, Metabolic Diseases, and Angiology, Faculty of Health Sciences in Katowice, Medical University of Silesia, Ziolowa 45/47 St., 40-635 Katowice, Poland

**Keywords:** heart failure, vitamin D, vitamin D deficiency, cardiovascular diseases, mortality

## Abstract

Heart failure (HF) remains a global health burden with high morbidity and mortality, despite significant pharmacological advances. Vitamin D deficiency (VDD) is commonly observed in HF patients and may exacerbate disease progression through various pathophysiological mechanisms, including activation of the renin–angiotensin–aldosterone system, inflammation, oxidative stress, and impaired calcium homeostasis. While vitamin D (VD) supplementation may positively influence surrogate markers in selected patient groups—particularly those with reduced ejection fraction or severe vitamin D deficiency—its effect on primary endpoints such as mortality or HF-related hospitalization varies significantly across studies and patient populations. As a result, while VD supplementation may benefit VD-deficient HF patients, current evidence does not support routine administration across the whole HF population. It is still a matter of debate whether VDD belongs to prognostic markers of worse outcomes in HF or is instead their potential cause. Therefore, the clinical utility of VD in HF management remains underexplored. This review aims to assess the evidence regarding vitamin D status and its supplementation in the context of HF, with a focus on different HF phenotypes: reduced (HFrEF), mildly reduced (HFmrEF), and preserved ejection fraction (HFpEF). The aim is to synthesize findings from novel observational studies, randomized controlled trials, and meta-analyses that shed light onto this intricate relationship and may be valuable in everyday clinical practice.

## 1. Introduction

Heart failure (HF) is a severe health problem worldwide, posing a major challenge to modern medicine. The prevalence of the disease is constantly increasing, mainly due to an aging population and an increasing average life expectancy [1]. The treatment with β-receptor blockers, angiotensin-converting enzyme inhibitors, angiotensin II receptor blockers, and mineralocorticoid receptor antagonists (MRAs) reduce cardiovascular events and improve prognosis in patients with HF with reduced ejection fraction (HFrEF), while sodium/glucose cotransporter 2 (SGLT2) inhibitors reduce hospitalization and mortality risk in patients with HF with preserved ejection fraction (HFpEF) and HFrEF [2,3,4]. Despite the development of therapy, patients with HF are still at high risk of death, development of other comorbidities, and frequent hospitalizations [5,6]. For this reason, there is a significant need for additional therapeutic strategies to further improve the prognosis of patients with HF.

Vitamin D deficiency (VDD) is a global issue, with an estimated prevalence of 40.4% among the population, and severe VDD affecting 13% of people in Europe [7]. The optimal level of 25-hydroxyvitamin D (25(OH)D) is still a subject of debate and varies depending on the recommendations of different societies. The Institute of Medicine (2010) [8] panel concluded that a serum 25(OH)D level of 20 ng/mL (50 nmol/L) is generally sufficient for bone and overall health in the general population, while the Endocrine Society (2011) [9] guidelines recommended levels above 30 ng/mL (75 nmol/L) to optimize vitamin D (VD) effects on calcium, bone, and muscle metabolism. However, according to the latest Endocrine Society guidelines from 2024, there is no clear evidence defining an optimal target level of 25(OH)D for disease prevention, suggesting against routine testing in those without conditions altering vitamin D physiology. In the diagnosis of vitamin D deficiency, different metabolites are used. Currently, the measurement of total 25-hydroxyvitamin D (t25(OH)D) is the primary method for assessing vitamin D status [10]. Serum 25(OH)D concentrations reflect vitamin D intake and endogenous production [11]. T25(OH)D is defined as a total of 25(OH)D metabolites, both in its free and protein-bound forms [12]. The concentration of 1,25-dihydroxyvitamin D (1,25(OH)2D) in blood is about three times lower compared to 25(OH)D, which makes measurement procedures difficult. Measurement of 1,25(OH)2D is useful in evaluating patients with unexplained hypercalcemia, sarcoidosis, granulomatous diseases, rickets, tumor-induced osteomalacia, and hyperparathyroidism [13]. The ability to measure 24,25-dihydroxyvitamin D (24,25(OH)2D) is a useful tool to identify individuals with genetic 24-hydroxylase defects, especially when 25(OH)D is detected in parallel [14]. T25(OH)D tests have limitations in cases of protein deficiency during liver diseases or loss in cases of nephrotic syndrome. In such cases, it may be beneficial to determine the free fraction of 25(OH)D (f25(OH)D) [15]. Only the free form of 25(OH)D can pass the lipophilic cell membrane, and it is believed to reflect biological activity more adequately than t25(OH)D. However, no clinical guidelines exist for the use of f25(OH)D [16].

**Figure 1:**
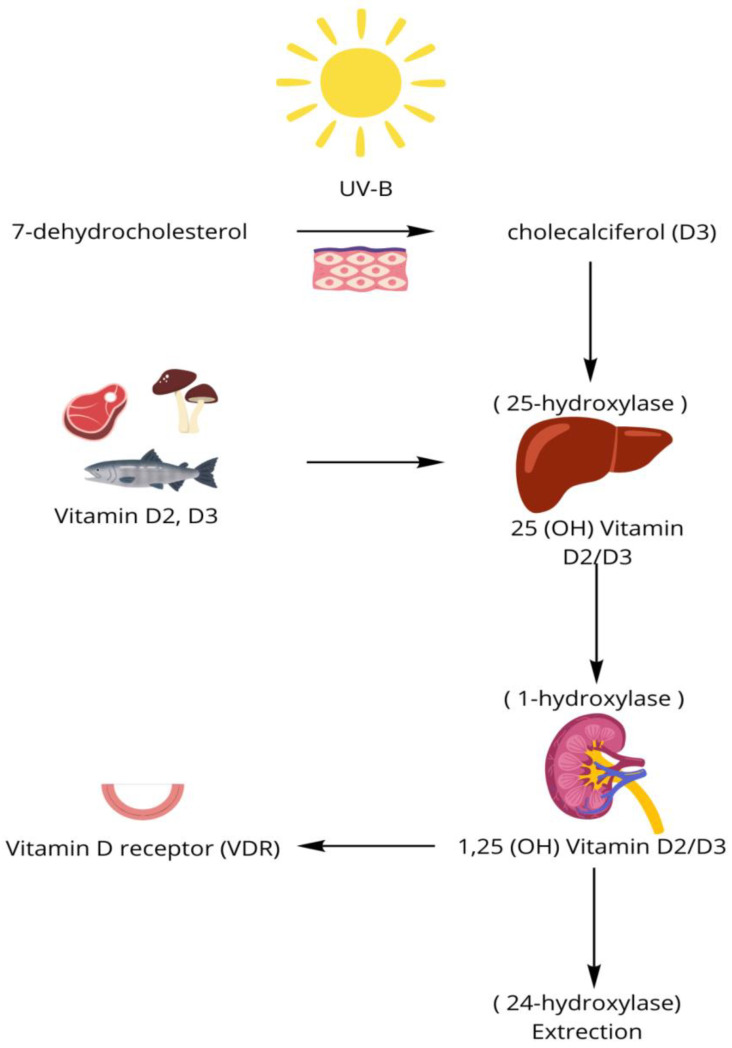
Biosynthesis process of vitamin D.

Few foods naturally contain vitamin D, making dermal synthesis through ultraviolet-B (UVB) radiation the primary source, responsible for approximately 90% of the body’s vitamin D supply. Cholecalciferol (vitamin D3) is derived from animal sources (such as meat, fish, and eggs), while ergocalciferol (vitamin D2) is found in plants and mushrooms. Under the influence of UV-B rays from sunlight, 7-dehydrocholesterol in the skin is converted into cholecalciferol [17]. Both vitamins D3 and D2 are biologically inactive and require further enzymatic conversion to become their active forms. In the liver, ergocalciferol and cholecalciferol are hydroxylated to calcidiol and calcifediol, respectively, which are the major forms of circulating vitamin D. In the kidneys, the 1-alpha hydroxylation reaction leads to the formation of ercalcitriol and calcitriol, with calcitriol being the most active form of vitamin D [18]. The level of VD is regulated by 24-hydroxylase, which leads to the inactivation of active forms and their excretion. Vitamin D and metabolites are carried in the blood mainly bound with vitamin D-binding protein (DBP) and albumin, with a small percentage transported in free form [15]. 1,25(OH)2D binds to the vitamin D receptor (VDR), which is located in the nucleus of most cells in the body. This process is crucial for many biological functions of vitamin D, such as regulating calcium and phosphate metabolism, bone metabolism, and the immune system [19]. The simplified biosynthesis is presented in Figure 1.

The impact of VD on the development of various diseases has been a frequently discussed topic in recent years. Multiple studies have investigated the relationship between vitamin D deficiency and cardiovascular diseases, including heart failure, hypertension, and atrial fibrillation [20,21]. VD deficiency is commonly observed in patients with HF, though its prevalence and clinical impact may differ across subtypes such as HFrEF and HFpEF, and depend on demographic factors including age, sex, and comorbidity profile [22]. Low vitamin D levels activate the renin–angiotensin–aldosterone system, generate inflammatory responses, and contribute to endothelial dysfunction [23]. However, existing studies have presented conflicting findings on the role of low serum 25(OH)D levels as a prognostic marker in patients with heart failure. This review seeks to compile findings from previous studies, analyze the results, and determine whether vitamin D supplementation improves outcomes in patients with heart failure.

## 2. Pathophysiology

According to the existing literature, VDD may be linked to HF; however, due to the paucity of data, further research is required in order to provide an in-depth insight into this intriguing relationship. Since the discovery of vitamin D, knowledge about its functions has advanced. The first and key role of vitamin D in human health was discovered by linking its deficiency with the development of rickets in children. For years, this led to the belief that its role was limited solely to musculoskeletal health [24]. The VDR was primarily discovered in tissues involved in the regulation of calcium and phosphate homeostasis, including the intestine, bone, kidney, and parathyroid glands [25]. However, the presence of VDR and alpha-hydroxylase in extraosseous tissues indicated that vitamin D might have physiological roles in other systems [26]. Studies on animals prove the existence of vitamin D receptors in ventricular cardiomyocytes and fibroblasts as well as in blood vessels [27,28,29]. Vitamin D can be activated extra-renally in tissues expressing 1α-hydroxylase. Cultured rat cardiomyocytes and cardiac fibroblasts exhibit 1α-hydroxylase expression in both the mRNA and protein levels. Additionally, in vitro studies have shown that endothelial and vascular smooth muscle cells locally produce 1,25(OH)2D from a labeled 25(OH)D substrate [30]. There is strong evidence for the specific role of vitamin D in the functioning of the cardiovascular system.

**Figure 2:**
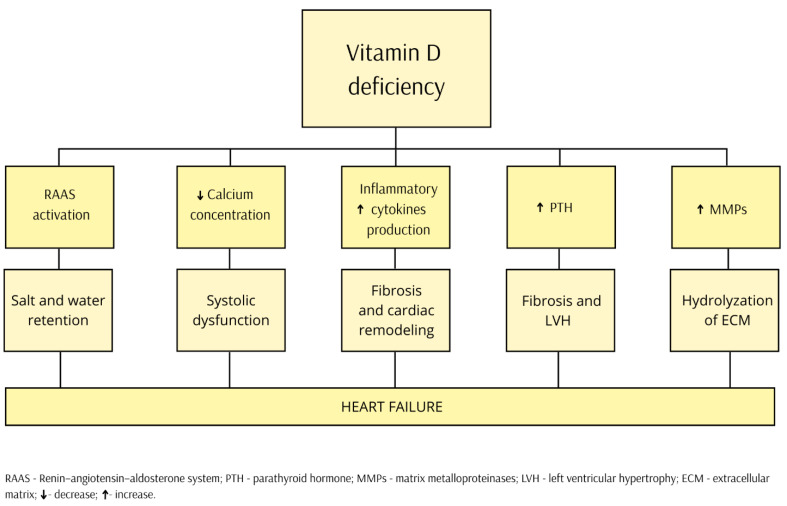
The pleiotropic effects of vitamin D deficiency, potentially affecting heart failure development.

The relationship between vitamin D deficiency and heart failure involves multiple mechanisms. Low vitamin D concentrations activate the renin–angiotensin–aldosterone system (RAAs), leading to sodium and water retention. Elevated aldosterone levels contribute to myocardial fibrosis and enhanced cardiac remodeling [31]. Vitamin D deficiency causes increased levels of oxidative stress by stimulating the production of proinflammatory cytokines such as interleukin-8 (IL-8) and tumor necrosis factor alpha (TNF-α). VD inhibits downregulation of nf-kb and causes decreased production of IL-6, -12, interferon- γ, and TNF-α [32]. The controversial issue, however, is whether vitamin D reduces inflammation or inflammation lowers 25(OH)D concentrations [33]. Calcium plays a key role in the contraction of cardiomyocytes. In vitamin D deficiency, calcium levels are reduced, which impairs cardiac contractility, leading to systolic dysfunction. 1,25(OH)2D stimulates calcium influx into cardiomyocytes via voltage-gated calcium channels, which produces a positive chronotropic effect [34]. Calcium deficiency leads to increased parathormone synthesis and secondary hyperparathyroidism, which causes myocardial fibrosis and hypertrophy as well as negatively affecting left ventricular systolic function [35]. Experimental studies have also shown the effect of vitamin D on extracellular matrix turnover with the activation of tissue inhibitors of metalloproteinases (MMPs), suggesting an antifibrotic and cardioprotective effect of vitamin D on cardiac remodeling. In the study, multipotent mesenchymal cells induced in a fibrotic phenotype after exposure to vitamin D metabolite exhibit decreased production of collagen and increase in production of metalloprotease-8 [36]. Preclinical studies indicated that the active form of VD—calcitriol—may stop the hypertrophy and proliferation of cardiomyocytes and, therefore, reduce the natriuretic peptides’ release [37]. The pleiotropic effects of VDD potentially affecting HF development are presented in Figure 2.

## 3. Vitamin D Deficiency

Patients with HF are at a higher risk of malnutrition, potentially leading to micronutrient deficiencies, consisting of not only VD but also minerals such as selenium. The attributable reasons are lack of appetite combined with poor dietary habits, increased metabolic demands, and malabsorption being a probable consequence of bowel wall edema [6]. Moreover, patients with HF are more often burdened with exocrine pancreatic insufficiency, which can be a coexisting condition in one in ten patients [38]. This may furtherly translate into deficiency of fat-soluble vitamins (A, D, and E) and fat-soluble minerals, such as selenium and zinc; however, it has not been confirmed by the existing studies yet [39].

In a study by Oliviera et al., which embraced 243 patients living in Brazil, from whom 161 were burdened with HF and recruited during hospitalization due to decompensation, patients with HF were at a higher risk of VDD (9.8% of patients with HF) and the overall serum 25(OH)D level was lower compared to the control group. Female sex and diabetes mellitus were factors predisposing to VDD. The differences between sexes may be explained by hepatic vitamin D-binding protein being regulated by sex steroid hormones, which declined in postmenopausal women [40]. Interestingly, living in a tropical climate does not guarantee sufficient protection against VDD [41]. Another study involving patients with HF living in a tropical country, Brazil, was carried out by Dantas-Komatsu et al. Approximately 7% of patients with HF were diagnosed with VDD, with women and patients with a higher New York Heart Association (NYHA) class being especially at risk. The discrepancies between VDD prevalence in tropical climate can be caused by differences in sun exposure, use of sunscreen, geographical latitude, and, therefore, various angles of solar radiation [40].

Skin pigmentation has been associated with variations in vitamin D synthesis and metabolism, which may influence HF risk differently in ethnically diverse populations. For example, in the Jackson Heart Study, Black participants showed a stronger association with higher 25(OH)D levels and reduced HFpEF risk than in predominantly White cohorts [42]. Higher 25(OH)D serum levels were also associated with inhibited LV concentric remodeling [42].

Moreover, VD hypovitaminosis is more often prevalent in patients with HF of ischemic aetiology, suggesting the relationship between VD and atherosclerosis [40]. It seems that VD and atherosclerosis represent a U-shaped relationship, with both deficiency and excess of VD bearing a negative impact. High VD levels (>50 ng/mL) were associated with a higher CV-related mortality. On the other hand, VD seems to be responsible for slowing down atherosclerosis progression. The pathophysiological mechanisms behind this are complex and embrace a few possible pathways. Firstly, VD has an anti-inflammatory effect, represses endothelial dysfunction by enhancing nitric oxide production, and reduces oxidative stress. Secondly, by promoting the expression of genes involved in cholesterol efflux, VD hinders the creation of foam cells responsible for atherosclerosis progression. This, combined with lipid metabolism modulation, results in higher plaque stability [43].

The optimal dosage of VD in HF was investigated in a recent meta-analysis by Chunbin et al., which covered the VD supplementation range from 2000 IU/day to 50,000 IU/week orally in patients with HF, regardless of LVEF. The meta-analysis showed no association between VD supplementation and changes in LVEF, 6 min walking test, and concentration of NT-pro BNP compared to the placebo group. Nevertheless, a dose-response analysis revealed a link between supplementation of 4000 IU/day of VD and improvement in LVEF, which indicated that it can be potentially beneficial for patients with HF [44]. Still, VD administration in HF without VDD has currently no scientific justification.

## 4. The Complicated Relationship

The growing number of studies on VD and HF highlights substantial heterogeneity in the study design, patient selection (e.g., LVEF categories, baseline VD status), outcome definitions, and dosing regimens, making it challenging to draw uniform conclusions. Therefore, it seems that recent studies deliver more questions than answers due to the inconsistent or even contradictory results, underlining the sophisticated nature of this topic. The most recent research studies on this matter are summarized below.

A Mendelian randomization study by Gao et al., which embraced 47,309 patients with HF, regardless of the LVEF, showed a linear association between VD serum concentration and HF risk [45]. This finding is in line with research by Ford et al., which revealed that VD supplementation in the elderly (≥60 years old) was beneficial in terms of HF risk reduction [46].

However, a study by de Oliveira et al., which embraced patients with HFrEF, HF with mildly reduced ejection fraction (HFmrEF), and HFpEF, found no association between VDD and HF-related variables, such as left ventricular ejection fraction (LVEF) or NYHA class in patients hospitalized due to HF decompensation [41]. This stands in contrast to the meta-analysis of randomized controlled trials by Zhao et al., which embraced 465 patients, who were in NYHA functional class ≥ II or with LVEF ≤ 40%, and demonstrated decrease in LVEDD and increase in LVEF in patients who were administered VD. Those results were particularly visible in subjects with reduced ejection fraction (EF). The potential role of VD supplementation in ventricular remodeling inhibition and cardiac function improvement requires further investigation [47].

Another research by Szabo et al. investigated the relationship between VD and LVEF in HFrEF and HFmrEF. Lower 25(OH)D levels were present in patients with severely reduced LVEF [48]. In a study by Mohanty et al., which embraced patients with congestive heart failure, the participants with VDD were administered 60,000 IU of cholecalciferol once a week for 12 weeks, apart from conventional pharmacotherapy for HF. Compared to the control group, patients receiving VD experienced decrease in NT-pro BNP, slight LVEF improvement, as well as left ventricular end-diastolic dimension (LVEDD) and left ventricular end-systolic dimension (LVESD) reductions. While no association was found between baseline 25(OH)D serum levels and LV functions and biomarkers, it seems that patients with congestive HF may benefit from VD supplementation. Trials with longer follow-up periods are required to provide information on the influence of VD supplementation on long-term prognosis [39].

A novel study on the relationship between vitamin D (VD) and congestive heart failure (HF) was conducted by Sun et al., who investigated the association between VD, magnesium, and congestive HF. Patients with low magnesium and VD levels were found to be at a higher risk of all-cause mortality and cardiovascular (CV)-related mortality. It is suggested that both VD and magnesium supplementation may have a combined effect in improving the prognosis of patients with congestive HF [49]. Nevertheless, due to the abundance of inconsistent results in studies investigating the relationship between 25(OH)D serum concentration and cardiac structure and function in HF, HF itself is not an indication of VD supplementation [50].

Herrmann et al. investigated the role of both 25(OH)D and 24,25(OH)2D in CV health and mortality in a prospective observational study and included participants with HF, regardless of LVEF. Patients with HF had a lower concentration of both forms of VD, with the highest deficiency being expressed particularly in patients with HFrEF. In addition, NT-proBNP concentrations were also higher in patients with lower 25(OH)D and 24,25(OH)2D. This research is in line with above-mentioned studies, confirming the relationship between low VD concentration and higher CV-related mortality [50]. It seems that higher CV-related mortality in VDD may be linked to higher frequency of thrombotic events, such as stroke and venous thromboembolism [51]

Interesting results were obtained in a study by Pirrotta et al., which analyzed the association between 25(OH)D serum levels and all-cause mortality, CV-related mortality, and HF hospitalization among elderly patients. It showed no relationship between VD and HF hospitalization or all-cause mortality; however, low 25(OH)D levels were attributable to heightened CV-related mortality. Noteworthy, VD deficiency is associated with osteoporosis and increased risk of bone fractures. While hip fractures increase the mortality rate in HF patients due to prolonged immobilization and muscle tone reduction, the relationship between bone mineral density (BMD) and HF remain vague. Few studies suggested a potential association between lowered BMD and higher HF risk or increased mortality; however, more research is needed to establish this link [52].

A group of patients, which need to be set apart, are people with left ventricular assist devices (LVAD). They are at a high risk of infection and sepsis, which contribute to the most common causes of death in this population [53]. VD deficiency may put those patients even at a higher risk of driveline infection, which was investigated in a study by Zittermann et al. However, the research was conducted on a relatively small group of patients (154 people) and more evidence is needed to establish the association [54]. The aforementioned results stand in line with prior research by Obeid et al., where low levels of 25(OH)D were associated with higher postoperative driveline infection risk and readmission rate [55].

Interestingly, a meta-analysis by Barbarawi et al., which embraced 21 randomized clinical trials and 83,291 patients, found no association between VD supplementation in HF and reduced risk of major adverse cardiovascular events, cardiovascular disease-related mortality, and all-cause mortality. This meta-analysis is fraught with a certain limitation since it included trials in which cardiovascular disease (CVD) was not the primary end point. That may have led to underestimation of CVD events and CVD-related deaths [56]. VITAL-HF, a substudy of VITAL trial, investigated the influence of VD and omega-3 fatty acids supplementation on HF-related hospitalization, regardless of the LVEF. During the 5-years follow-up, the study showed no differences between VD and placebo in terms of incidence of HF hospitalization. Similar results were obtained with omega-3 fatty acids [57].

More and more research papers investigated the link between VD and HF. However, the majority of them did not differentiate between HF types regarding the LVEF (HFpEF, HFmrEF, HFrEF). The paucity of data deters, especially, HFpEF, which should be distinct from other HF types due to different pathophysiology and clinical character.

## 5. HFpEF

Patients burdened with HFpEF may comprise up to 50% of heart failure cases and create a heterogenous group, with aging being the most common cause. Other risk factors are T2DM, hypertension, female sex, obesity, iron deficiency, and sleep apnea. The variety of HFpEF phenotypes makes it even harder to conduct research and choose the adequate study group. However, this condition should not be underestimated; the morbidity rates in HFpEF are comparable to those in HFrEF. The role of vitamin D supplementation in HFpEF is terra incognita [58]. One observational study by Nolte et al. investigated the role of 25(OH)D levels in HFpEF. Lower 25(OH)D concentrations were associated with higher prevalence of HF symptoms such as oedema, fatigue and nocturnal cough, and higher functional NYHA class. Interestingly, lower 25(OH)D concentration was also a predictor of increased values of NT-proBNP and increased rate of cardiovascular hospitalizations; however, it did not influence the 5-year mortality. The rate of VDD in the studied group was as high as 33.4% [59].

Whether VDD puts the patients at a higher risk of HFpEF development is questionable. In a study by Fall et al., echocardiography was performed on 70-year-old patients combined with 25(OH)D concentration measurement. Higher 25(OH)D concentration was associated with decreased LVEDD and increased measures of systolic function, such as fractional shortening and ejection fraction. However, during the 5-year follow-up, no significant links between the LV measures and 25(OH)D concentration were observed, which puts the above-mentioned results in question [60]. Moreover, another study investigating the association between 25(OH)D concentration and echocardiographic parameters in a population-based cohort found no link between these two variables [61].

In conclusion, the inconsistent results of existing studies make it harder to establish a consensus in guidelines on HF treatment. Whether patients without VDD could benefit from supplementation in HF remains questionable. Moreover, the paucity of research on VD differentiates between the particular HF categories, grouped according to LVEF. This stresses the importance of further research on this matter.

## 6. HFrEF

The most common etiology of HFrEF is ischemic heart disease, comprising up to 50% of cases. Other possible underlying conditions encompass valvular heart diseases, cardiomyopathies, and viral infections. Despite the huge progress in the field of HFrEF patients’ management and treatment, it still remains a serious condition with a high 5-year mortality. After hospitalization for HFrEF, the 5-year survival is as low as 24.7% [58,62]. The prevalence of VDD ranges from 56% to 83% in patients with HFrEF [63].

It is suggested that VDD translates to impaired LV structure and function, being also responsible for reduced functional capacity and elevated levels of cardiac biomarkers. Lower 25(OH)D concentrations were associated with worse outcomes of the 6 min walking test and lower peak oxygen uptake in a cardiopulmonary test. Interestingly, no association was found between 25(OH)D concentration and functional NYHA class in HFrEF patients. VDD may be also linked to increased rate of HF readmission and higher mortality. However, the confirmation of VD influence on the significant HF endpoints, such as HF rehospitalization, CV mortality, and all-cause mortality, is hard to find in randomized controlled trials. While it may positively influence secondary endpoints, such as B-type natriuretic peptide (BNP) concentration, parathormone (PTH), and LVEF, all in all, VD supplementation did not prove to be superior to conventional treatment regarding LVEF, NT-proBNP, and six-minute walking distance (6MWD) [63].

The above-mentioned research results pose a question on the relationship between VD and HFrEF. It remains disputable if VDD is a potential cause of worse outcomes or as a prognostic marker resulting from malnutrition, reduced outdoor activity, and concomitant chronic kidney disease. Undoubtedly, VDD should be actively sought after and treated; however, the cause-and-effect relationship between VDD and HFrEF is not yet established [63].

## 7. Chronic Kidney Disease

While VD supplementation in chronic kidney disease (CKD) treatment belongs to a standard of care, its role in patients burdened with both HF and CKD seems to be sophisticated [64]. HF and CKD are of increasing prevalence in the elderly population and share similar risk factors such as diabetes mellitus or hypertension. Moreover, one condition worsens the outcomes and accelerates the progression of the other, creating a vicious cycle. Therefore, the coexistence of HF and CKD belongs to clinical challenges and requires a multidisciplinary approach [65]. While HF pharmacotherapy (SGLT2 inhibitors, RAAs inhibitors, MRAs) is proven to slow down the CKD and macroalbuminuria progression with proteinuria improvement, the concomitance of HF and CKD remains a serious clinical problem. Micro- and macroalbuminuria, which are common in patients with CKD related to CVD, heighten the risk of HF hospitalization and mortality. Therefore, there are premises to treat albuminuria as one of CV risk markers [66]. Szabo et al. found a positive correlation between serum 25(OH)D and albumin levels. It seems that VDD may predispose to hypoalbuminemia occurrence over the course of HF and coexisting CKD. Therefore, it is suggested that VD supplementation may add to hindering the CKD progression and improve HF patients’ prognosis by increasing the serum albumin levels [48].

A study paper by Raimer et al., embracing patients with CKD and estimated glomerular filtration rate (eGFR) 30–60 mL/min/1.73 m^2^, found an association between higher 25(OH)D levels and lover risk of MACE and HF and CV-related death. While VDD was common in ca. 80% of the study group, it seems that VD supplementation may translate into better outcomes when heightening the 25(OH)D serum levels to the optimum [67]. Interestingly, the CRIC study found no link between serum concentrations of 25(OH)D and 1,25(OH)2D and risk of CV disease in patients with no dialysis CKD [68]. Interesting results were brought up by Arroyo et al. in a study involving patients after a kidney transplant and those being on a waitlist. The study investigated the levels of serum epimeric vitamin D (epi-25(OH)D), which can make up to more than 50% of total serum 25(OH)D and is positively correlated with exercise capacity, skeletal muscle mass, and strength. The epi-25(OH)D serum levels were increasing after kidney transplantation and were declining in patients on the transplant waitlist, and those observed alterations were longitudinally associated with peak oxygen consumption. Moreover, low levels of epi-25(OH)D influenced negatively the results of cardiopulmonary exercise testing, resulting in impaired cardiovascular functional capacity. This stresses the importance of further investigating the role of epi-25(OH)D in patients with advanced CKD, which may become one of potential treatment options [69].

On the contrary to the above-mentioned studies, research by Cheng et al. investigated the influence of free 25(OH)D serum concentration. Its 2-fold higher concentration was associated with lower risk of HF but higher risk of kidney function decline. The negative influence on kidneys was absent when taking into consideration solely the 25(OH)D concentration. The current evidence on free VD and kidney function decline is scarce and the existing research on 25(OH)D concentration and kidney function decline provide inconsistent results [70]. Still, the importance of VD supplementation in HF patients with CKD is poorly supported due to the paucity of randomized controlled trials and heterogeneity of results in observational studies [64].

## 8. Metabolic Diseases

Patients with HF are usually burdened with a variety of concomitant diseases, which remain in a complex relationship. It is estimated that even 40% of patients with HF are affected by at least 5 non-cardiovascular comorbidities [64]. Diabetes, hypertension, dyslipidemia, and obesity do not only belong to the risk factors for HFpEF but may also result in ischemic heart disease, which leads to HFrEF [58]. VD is not without significance when it comes to metabolic diseases, as VD deficiency is associated with an increased incidence of type 2 diabetes mellitus (T2DM), hypertension, obesity, insulin resistance, and metabolic syndrome (MS) [43,71]. VD supplementation seems to be beneficial for patients with VDD by increasing peripheral insulin sensitivity and pancreatic β-cell function, while reducing the risk of T2DM complications such as diabetic retinopathy [71].

In a recent cohort study by Chen et al. involving 15,226 participants with T2DM, higher 25(OH)D serum levels were associated with a lower prevalence of HF in those patients [72]. Similar results were obtained by Tougaard et al. in a cohort study investigating the role of VD in type 1 diabetes mellitus (T1DM) and T2DM. VDD was associated with a heightened risk of HF in both T1DM and T2DM groups as well as MACE in the T1DM group. Interestingly, the research does not confirm the relationship between VDD and microvascular complications development [73]. In a study by de Oliveira et al., VDD was associated with higher HbA1c, total cholesterol and PTH blood levels, and lower eGFR [41]. All in all, patients with diabetes and VDD may additionally benefit from VD supplementation, especially in terms of diabetes-related complications progression [74].

VD supplementation also results in lipid profile improvement by reducing serum total cholesterol (TC), low-density lipoprotein cholesterol (LDL), and triglyceride (TG) levels [75]. This is due to modulating the activity of hepatic and lipoprotein lipase that VD positively influences the balance between LDL and high-density lipoprotein cholesterol (HDL). The link between hypertension (HT) and VD seems to be multifactorial, embracing inhibition of RAAs, promoting vasodilation via NO production, and regulation of calcium homeostasis. Patients with HT and VDD may benefit from VD supplementation because its deficiency is associated with a heightened risk of HT development [43].

The DO-HEALTH randomized controlled trial stands in contrast to the aforementioned studies. It was a three-year double-blind, randomized placebo-controlled trial with 2157 participants, who were administered with 2000 IU/day of VD or 1 g/day of omega-3 fatty acids or home strength exercise program for 3 times/week. VD administration was not associated with lipid profile improvements, lowering NT-proBNP concentrations, nor with MACE reduction. However, the study solely embraced participants without major chronic conditions, which may be the cause of results differing from other studies. Therefore, it seems that healthy patients will not necessarily benefit from VD supplementation in terms of CV health [49]. In a study by Khan et al., which summarized meta-analyses of randomized controlled trials on nutritional supplements and dietary intervention in HF, a Mediterranean diet was associated with lower HF risk with coenzyme Q reducing all-cause mortality in HF patients. Limited body of evidence supported the hypothesis that supplementation of thiamine, VD, iron, and L-carnitine positively influenced LVEF. However, lack of high-quality evidence necessitates further research and limits their clinical implementation for the time being [76].

## 9. Sarcopenia

Sarcopenia belongs to one of major comorbidities in HF with its mean prevalence being estimated at 34%, ranging from 10 to 68%, depending on the study [77]. The coexistence of a few contributing factors such as inflammation, malnutrition, metabolic disturbances, and low physical activity is the cause of sarcopenia, creating a vicious cycle with concomitant HF, as HF worsens sarcopenia-associated complications such as frailty, osteoporosis, and cachexia. On the other hand, sarcopenia further worsens the quality of life in HF patients [77,78]. However, according to an up-to-date systematic review and meta-analysis, the significance of sarcopenia in HF prognosis is not yet established [79]. VD has a significant impact on muscle metabolism and function. Its deficiency translates into muscle strengths reduction and higher falling hazard because of fatigue. Existing studies demonstrate inconsistent results in terms of sarcopenia prevalence and VDD; however, most studies support the existence of vital relationship between them, as VDD belongis to independent sarcopenia risk factors [80]. According to existing research, VD supplementation does not influence muscle mass; however, it may increase muscle strength and prevent muscle loss [81,82]. All in all, due to conflicting results obtained in VD supplementation trials in sarcopenia, the benefits of VD administration seem to be limited [79]. The results of the conducted clinical trials suggest that supplementing both proteins and VD can positively alter everyday functioning [81,83]. Paucity of data exists on VD influence on sarcopenia and heart failure population of patients. A novel study by Nagaoka et al. involving 461 participants, suggest the existence of a U-shaped relationship between sarcopenia probability in patients with HF and 25(OH)D serum levels. Further research is needed to establish the optimal range of 25(OH)D levels and to determine the clinical significance of this finding [84].

## 10. Conclusions

The heterogeneity of existing studies—including differences in population demographics, heart failure phenotypes, baseline vitamin D status, supplementation regimens, and endpoints—complicates direct comparison and generalizability of findings. Stratification by these variables is essential for future research to elucidate subgroups that may derive the most benefit from VD supplementation.

In general, VD should be administered to patients with its deficiency, without concerns about heightening the CV risk [85]. However, the criterion of implementing VD supplementation is still undefined; it may be based on LVEF, PTH levels or 25(OH)D serum level. However, further research is needed [47]. Moreover, it is still debatable whether VD is a marker of general health and CV risk factors or rather has direct impact on CV heath [50]. This poses a question whether patients with HF and without VD deficiency will benefit from VD supplementation. The discrepancies between existing research papers emerge from different biomarkers of VD status and VDD cutoff values, no differentiation between HFrEF, HFmrEF, and HFpEF, diverse LVEF in the studied population, and various cholecalciferol supplementation schemes embracing doses and administration intervals [48,64]. Given the variability in baseline vitamin D status, heart failure etiology and subtype, as well as heterogeneity in study populations and dosing protocols, current evidence does not support the routine VD supplementation in all patients with HF. Instead, a personalized approach, targeting patients with documented deficiency and certain HF phenotypes (e.g., HFrEF), may be more appropriate. It is also worth stressing that active vitamin D compounds, such as 1,25(OH)2D, may differ from VD supplements in terms of potential risks and benefits [64]. While VD may have a positive impact on immune regulation and osteoporosis, its use in HF is still a subject of research, with promising results in animal studies, exhibiting influence on cardiac remodeling and function [50,85]. However, the potential cardioprotective influence of VD does not currently seem to be significant enough to provide additional benefits for HF patients on guideline-based treatment [63].

More randomized controlled trials with adequate observation periods are necessary to investigate the potential benefits of VD supplementation in patients with HF and without VDD, taking into consideration the influence on vital endpoints, such as HF rehospitalization, CV mortality, and all-cause mortality. Moreover, further research is required to establish optimal dosage and therapy monitoring.

## Data Availability

We used PubMed, SCOPUS, and ScienceDirect databases to screen articles for this review. We did not report any data.

## Abbreviations

The following abbreviations are used in this manuscript:

1,25(OH)2D	1,25-dihydroxyvitamin D, calcitirol
24,25(OH)2D	24,25-dihydroxyvitamin D
25(OH)D	25-hydroxyvitamin D, calcifediol
6MWD	six-minute walking distance
BNP	B-type natriuretic peptide
CKD	chronic kidney disease
CV	cardiovascular
CVD	cardiovascular disease
eGFR	estimated glomerular filtration rate
epi-25(OH)D	epimeric vitamin D3
f25(OH)D	free fraction of 25-hydroxyvitamin D
HbA1c	glycated haemoglobin
HDL	high-density lipoprotein cholesterol
HF	heart failure
HFmrEF	heart failure with mildly reduced ejection fraction
HFpEF	heart failure with preserved ejection fraction
HFrEF	heart failure with reduced ejection fraction
HT	hypertension
LDL	low-density lipoprotein cholesterol
LV	left ventricle
LVAD	left ventricular assist devices
LVEDD	left ventricular end-diastolic dimension
LVEF	left ventricular ejection fraction
LVESD	left ventricular end-systolic dimension
MACE	major adverse cardiovascular events
MMPs	metalloproteinases
MRAs	mineralocorticoid receptor antagonists
NO	nitric oxide
NT-proBNP	N-terminal pro-B-type natriuretic peptide
NYHA	New York Heart Association
PTH	parathyroid hormone
RAAs	renin-angiotensin-aldosterone system
SGLT2	sodium/glucose cotransporter 2
T1DM	type 1 diabetes mellitus
T2DM	type 2 diabetes mellitus
T25(OH)D	total of 25-hydroxyvitamin D metabolites
TC	total cholesterol
TG	triglycerides
VD	vitamin D
VDD	vitamin D deficiency
VDR	vitamin D receptor
